# The Utilization of Rice Husk as Both the Silicon Source and Mesoporous Template for the Green Preparation of Mesoporous TiO_2_/SiO_2_ and Its Excellent Catalytic Performance in Oxidative Desulfurization

**DOI:** 10.3390/molecules29163856

**Published:** 2024-08-14

**Authors:** Xiaoxue Liu, Lanfen Zhang, Jian Hu, Wei Zhang, Xiaorong Xiang, Huiqing Cheng, Li Qin, Hao Li

**Affiliations:** 1 College of Agriculture, Yangtze University, Jingzhou 434000, China; 2 College of Chemistry and Environmental Engineering, Yangtze University, Jingzhou 434023, China

**Keywords:** rice husk, incipient wetness impregnation, mesoporous TiO_2_/SiO_2_, oxidative desulfurization, recycling catalytic performance, Ti-peroxo species

## Abstract

In recent years, TiO_2_-based catalysts have received extensive attention from researchers for their excellent oxidative desulfurization (ODS) performances. In this paper, a series of mesoporous TiO_2_/SiO_2_ catalysts with different TiO_2_ loadings are prepared, using an incipient wetness impregnation method with agricultural waste rice husk as both the silicon source and mesoporous template and tetrabutyl titanate as the titanium source. The effect of different TiO_2_ loadings on the ODS performance of the samples is investigated, and the appropriate TiO_2_ loading is 2.5%. Compared with pure TiO_2_, the 2.5%TiO_2_/SiO_2_ sample exhibits high catalytic activity for oxidative desulfurization. This is, on the one hand, due to the high specific surface area and mesopore volume of the 2.5%TiO_2_/SiO_2_ sample. On the other hand, it is due to the uniform dispersion of TiO_2_ grains with an average diameter of 6.1 nm on the surface of the mesoporous SiO_2_ carrier, which greatly increases the active sites of the 2.5%TiO_2_/SiO_2_ sample, thus improving the catalytic activity of the sample. The recycling performances of the 2.5%TiO_2_/SiO_2_ sample are further investigated. The results show that, after fifteen cycles, the 2.5%TiO_2_/SiO_2_ sample still maintains high conversions of dibenzothiophene (99.8%) and 4,6-dimethyldibenzothiophene (99.7%) without deactivation. In addition, the 2.5%TiO_2_/SiO_2_ sample treated with TBHP aqueous solution is characterized by the technique of UV-Vis, and the Ti-peroxo (Ti-OO^t^Bu) species, the active intermediate for the ODS of bulky organic sulfides, is successfully captured. Finally, a possible reaction mechanism for the ODS process over the 2.5%TiO_2_/SiO_2_ sample is proposed.

## 1. Introduction

SO*x* gases emitted from the combustion of sulfides in fuel oil cause a series of environmental pollution problems [1]. Therefore, many countries around the world are adopting increasingly stringent requirements for the sulfur content in fuel oil, and China has relied on VIA standard gasoline (sulfur content < 10 ppm) since 1 January 2019 [2]. Among the various desulfurization technologies, oxidative desulfurization (ODS) is a desulfurization technology that first oxidizes thiophene sulfides in fuel oils into corresponding sulfoxide or sulfone compounds under the action of catalysts and oxidants, and then separates them from the system using polar solvents. This technology is considered as one of the effective methods for deep desulfurization due to its advantages such as mild reaction conditions, low input costs, and the easier removal of thiophene sulfides without affecting the octane number of fuel oils [3,4]. 

The commonly used catalysts for oxidative desulfurization mainly include polymetallic oxides [5] and their composites [6], titanium-containing molecular sieves [7,8], and transition metal oxides [9,10,11,12,13,14,15,16], etc. Among these catalysts, TiO_2_-SiO_2_ composites are favored by researchers for their excellent desulfurization performance [13,14,15,16]. Ji et al. [13] prepared amorphous TiO_2_-SiO_2_ composites with different TiO_2_ contents using a precipitation method. Among them, the 40%TiO_2_-SiO_2_ composite had a desulfurization rate of 78% in the ODS of gasoline with hydrogen peroxide–acetic acid as the oxidant. Liu et al. [14] synthesized amorphous mesoporous TiO_2_-SiO_2_ composites by introducing n-propyltriethoxysilane (PTES) into the synthesis system of titanium silicalite-1. Among them, the TS-0.2 (the PTES/Si molar ratio: 0.2) sample showed high catalytic activity and stability in the ODS of dibenzothiophene (DBT) and 4,6-dimethyldibenzothiophene (4,6-DMDBT) model oils. After five cycles, TS-0.2 still maintained high DBT conversion (99.0%) and 4,6-DMDBT conversion (99.8%) rates without deactivation. Bazyaria et al. [15] prepared microporous TiO_2_-SiO_2_ nanocomposites with different TiO_2_ contents by the sol–gel method. Among them, the TiO_2_-SiO_2_ (the TiO_2_ loading: 50 wt.%) had a desulfurization rate of over 99% in the ODS of DBT model oil with TBHP as the oxidant. However, the cyclic experiments were conducted only twice. Ghahramaninezhad et al. [16] synthesized a novel catalyst (TS-4/CM-β-CD) by combining carboxymethyl-functionalized β-cyclodextrin with TiO_2_/SiO_2_ nanoparticles under the action of cyanamid. Under the optimized reaction conditions, the TS-4/CM-β-CD catalyst achieved a DBT desulfurization rate of 99.6%. After three cycles, TS-4/CM-β-CD still maintained a high DBT desulfurization rate (99.0%). However, most of the above-mentioned TiO_2_-SiO_2_ composites were prepared using commercial tetraethyl silicate (TEOS) as the silicon source. The production process of TEOS is both expensive and environmentally unfriendly [17]. Therefore, in order to minimize the environmental and economic impacts of using TEOS, it is particularly important to find a green, inexpensive, and environmentally friendly silicon source.

Rice husk (RH) is an agricultural residue produced during rice processing. RH mainly contains 22.31% lignin, 36.11% cellulose, 19.45% hemicellulose, and 20.11% silica [18]. RH is rich in amorphous silica, which is a green silicon source that can replace commercially available TEOS. The pathway of using RH as a silica source for the preparation of SiO_2_-based catalytic materials is an economical and environmentally friendly approach, which is in line with the principles of Green Chemistry [12,19,20,21]. Recently, our team [12] prepared a series of mesoporous WO_3_/SiO_2_ catalysts with different WO_3_ loadings, using the incipient wetness impregnation method with RH as both the silicon source and mesoporous template and phosphotungstic acid as the tungsten source. Among them, the WS-923-15 sample showed good catalytic activity and stability in the ODS of DBT and 4,6-DMDBT model oils. As an extension of the above work, in this paper, a series of mesoporous TiO_2_/SiO_2_ catalysts with different TiO_2_ loadings are continued to be prepared by a similar method, using RH as both the silicon source and mesoporous template and tetrabutyl titanate (TBOT) as the titanium source. The catalytic performances of the TiO_2_/SiO_2_ samples in the ODS of DBT and 4,6-DMDBT model oils are studied, the recycling performances of the TiO_2_/SiO_2_ samples are investigated, and the catalytic mechanism of the ODS process is explored. 

## 2. Results and Discussion

### 2.1. Structural Characterizations

For the convenience of readers, the nomenclature of the catalysts is first explained. For example, in ‘TS-RH-2.5c’, ‘TS’ is the abbreviation for TiO_2_/SiO_2_ catalysts, ‘RH’ is the abbreviation for rice husk, ‘TS-RH’ denotes TiO_2_/SiO_2_ catalysts prepared using RH as feedstock, ‘2.5’ stands for the loading of TiO_2_, and ‘c’ stands for the regenerated sample (see Section 3.2 and Section 3.5 for details). 

**Figure 1:**
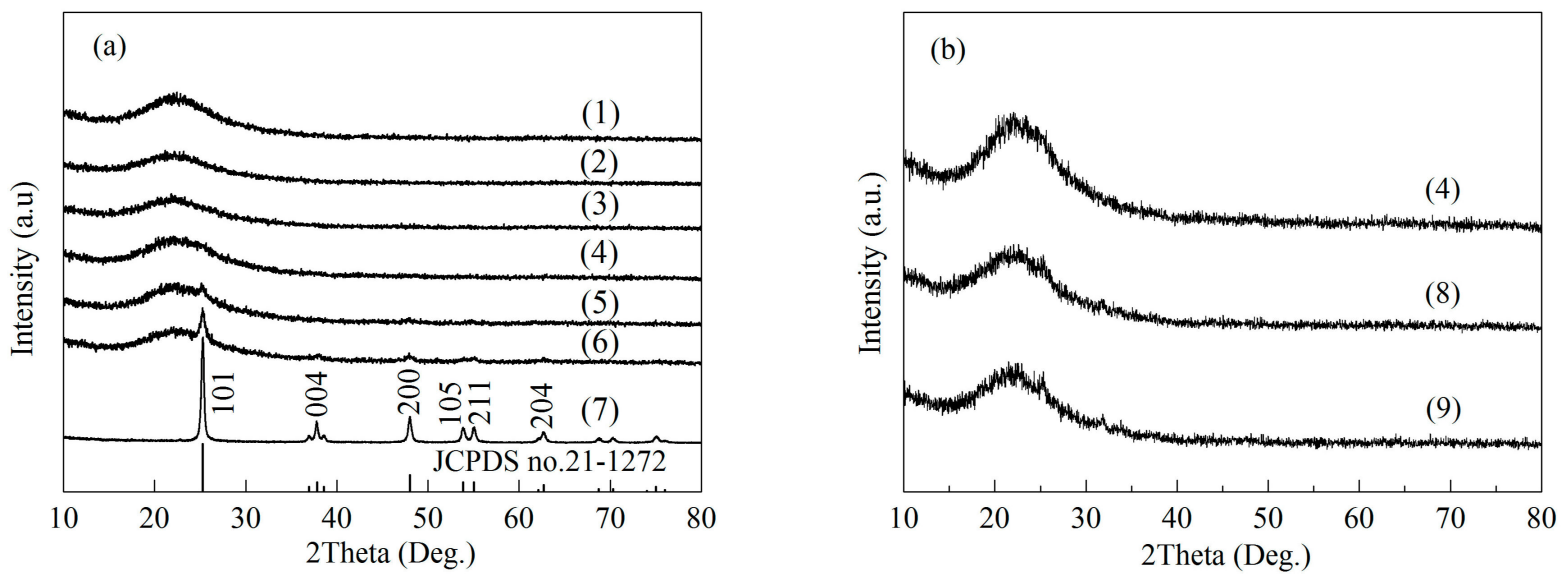
The XRD patterns of the SiO_2_-RH, pure TiO_2_, and TS-RH-x samples (**a**), and the fresh and regenerated TS-RH-2.5 samples (**b**): (1) SiO_2_-RH, (2) TS-RH-0.5, (3) TS-RH-1.0, (4) TS-RH-2.5, (5) TS-RH-5.0, (6) TS-RH-10, (7) pure TiO_2_, (8) TS-RH-2.5c regenerated from the 15th ODS reaction of DBT, and (9) TS-RH-2.5c regenerated from the 15th ODS reaction of 4,6-DMDBT.

Figure 1 shows the XRD patterns of the SiO_2_-RH, pure TiO_2_, TS-RH-x, and TS-RH-xc samples. As can be seen from Figure 1a, the SiO_2_-RH carrier has a broad and diffuse diffraction peak near 23.0°, indicating that it has an amorphous structure. Pure TiO_2_ exhibits diffraction peaks at 25.3°, 37.8°, 48.0°, 54.0°, 55.1°, and 62.9°, which correspond to the (101), (004), (200), (105), (211), and (204) crystal planes of the anatase phase of TiO_2_ (JCPDS no. 21-1272), respectively [22]. With an increase in TiO_2_ loading from 0.5 to 2.5%, the TS-RH-x samples still display a broad and diffuse diffraction peak near 23.0°, indicating that the samples have an amorphous structure and the TiO_2_ grains are highly dispersed on the surface of the SiO_2_-RH carrier. Continuing to increase the loading of TiO_2_, the samples start to exbibit the characteristic peaks of anatase TiO_2_. In addition, compared with the fresh TS-RH-2.5 sample (Figure 1b), the regenerated samples show an amorphous structure despite the appearance of a very weak diffraction peak of anatase TiO_2_ at 25.3°. 

**Figure 2:**
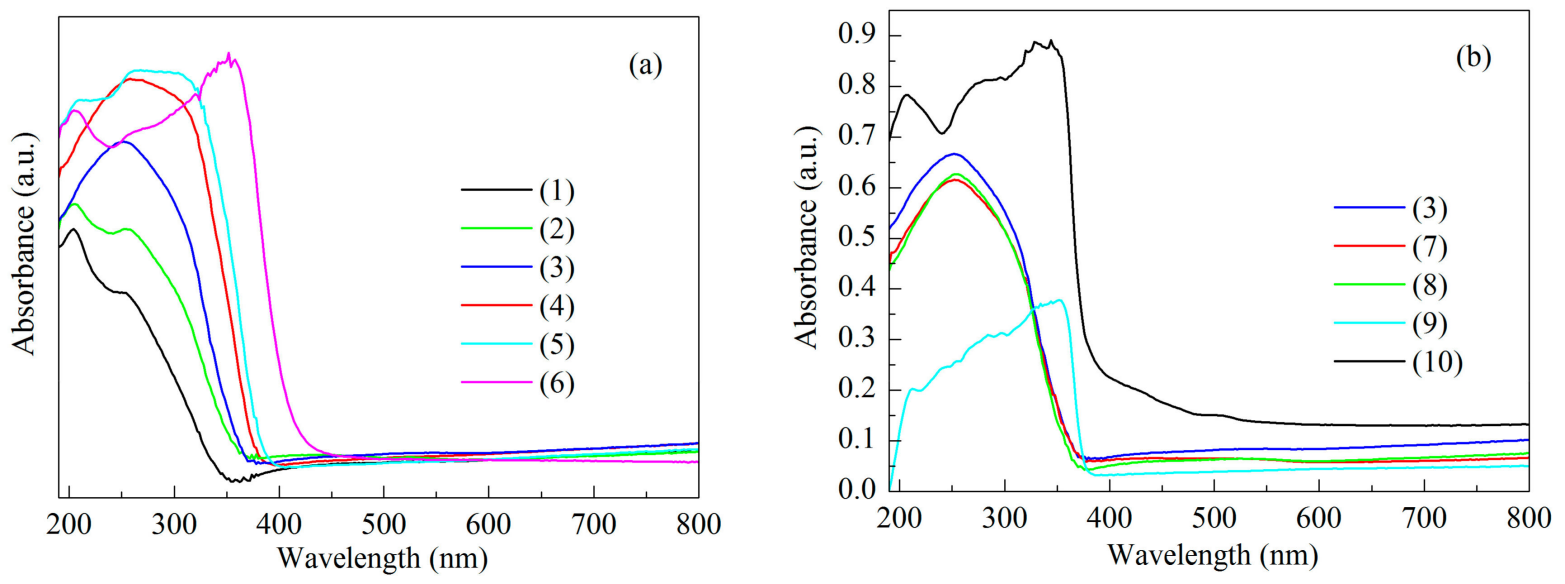
UV-Vis spectra of the pure TiO_2_, and TS-RH-x samples (**a**), and the fresh and regenerated TS-RH-2.5 samples, and oxidation products of DBT and 4,6-DMDBT (**b**): (1) TS-RH-0.5, (2) TS-RH-1.0, (3) TS-RH-2.5, (4) TS-RH-5.0, (5) TS-RH-10, (6) pure TiO_2_, (7) TS-RH-2.5c regenerated from the 15th ODS reaction of DBT, (8) TS-RH-2.5c regenerated from the 15th ODS reaction of 4,6-DMDBT, (9) oxidation product of DBT, and (10) oxidation product of 4,6-DMDBT.

Figure 2 shows the UV-Vis spectra of the samples. As shown in Figure 2a, the pure TiO_2_ has distinct absorption bands in the UV region (at 206, 261, and 352 nm) and no photo-response in the visible region [22]. TS-RH-0.5 displays three absorption bands at 205, 255, and 310 nm, corresponding to the tetra-coordinated titanium species, hexa-coordinated titanium species, and anatase-phase TiO_2_, respectively [14,23,24]. As the TiO_2_ loading increases from 0.5 to 2.5%, the absorption intensity gradually increases and the peak width gradually broadens. Moreover, the main absorption band redshifts from 205 to 256 nm. Continuing to increase the TiO_2_ loading, the redshift of the maximum absorption band is not large, the absorption intensity is gradually enhanced, and the peak width is gradually widened. The above results show that the strongest absorption band redshifts occur with increasing the TiO_2_ loading, indicating an increase in the coordination number of titanium species in the sample [23,24]. It has been shown that both tetra- and hexa-coordinated titanium species exhibit good catalytic oxidation activity in the presence of organic peroxides [24]. As shown in Figure 2b, compared to the spectra of the oxidized products, the TS-RH-2.5c samples are free of DBT and 4,6-DMDBT oxidized products after regeneration by calcinating. Compared with the fresh TS-RH-2.5 sample, the absorption intensity of the regenerated samples is slightly reduced, indicating that their TiO_2_ loadings are not reduced to a large extent after the recycle tests. 

**Figure 3:**
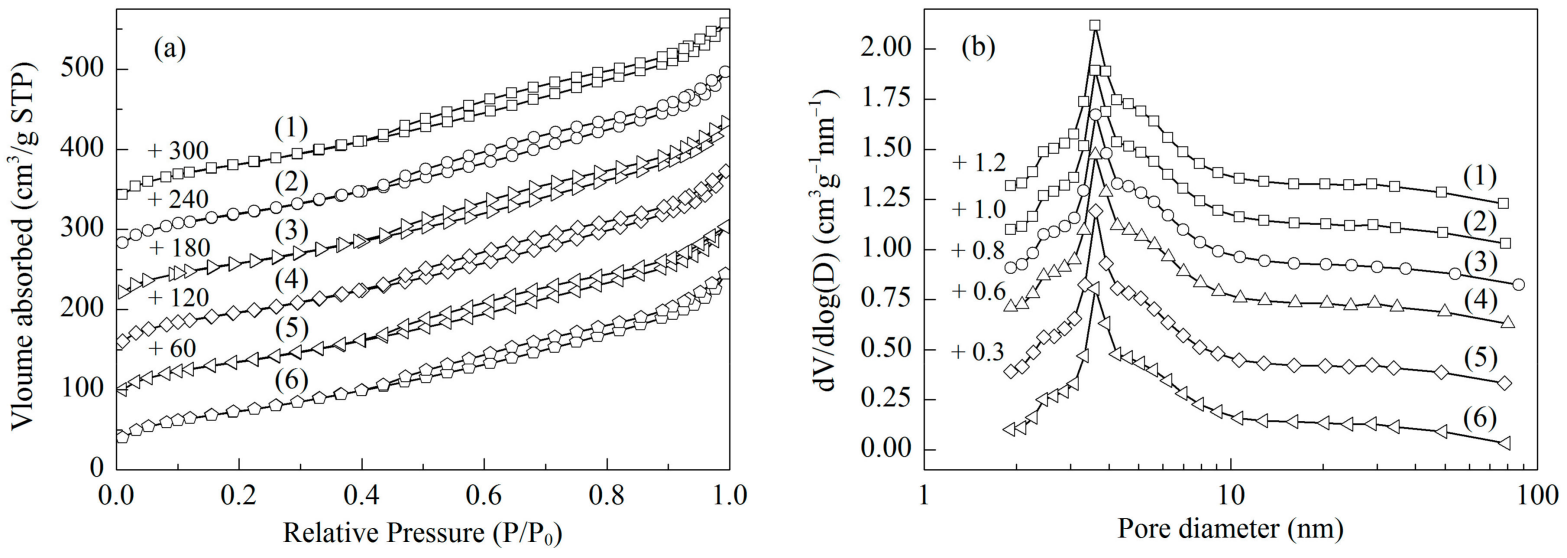

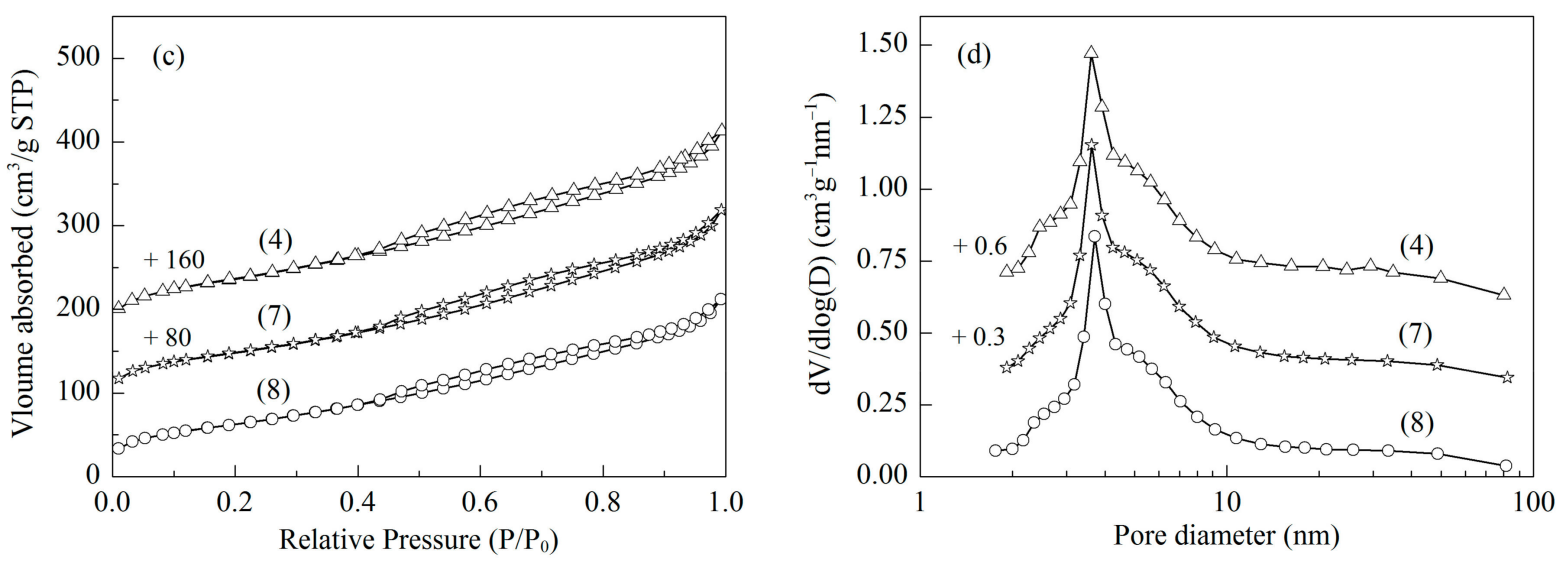
N_2_ adsorption–desorption isotherms (**a**,**c**) and pore size distribution curves (**b**,**d**) of the samples: (1) SiO_2_-RH, (2) TS-RH-0.5, (3) TS-RH-1.0, (4) TS-RH-2.5, (5) TS-RH-5.0, (6) TS-RH-10, (7) TS-RH-2.5c regenerated from the 15th ODS reaction of DBT, and (8) TS-RH-2.5c regenerated from the 15th ODS reaction of 4,6-DMDBT.

Figure 3 shows the N_2_ adsorption–desorption isotherms and pore size distributions of the samples. As can be seen from Figure 3a, both the SiO_2_-RH and TS-RH-x samples show typical type IV isotherms in the IUPAC classification, with a clear H4 hysteresis loop at P/P_0_ > 0.40, suggesting that the samples have a mesoporous structure. The isothermal shapes of the TS-RH-x samples are similar compared to the SiO_2_-RH, suggesting that they have similar pore structures. The pores in the SiO_2_-RH carrier are centrally distributed within the range of 1.9–78 nm, with the most probable pore size being 3.6 nm (Figure 3b). With an increase in the TiO_2_ loading from 0.5 to 10%, the pore distribution and the most probable pore size of the TS-RH-x samples do not change much, while the S_BET_ and V_meso_ decrease slightly, indicating that the TiO_2_ grains are well dispersed on the surface/pores of the SiO_2_-RH carrier and do not block its pores. Compared with the pure TiO_2_, the TS-RH-x samples have a larger S_BET_ and V_meso_, which is conducive to the easy access of bulky substrates to the active sites, overcoming the diffusion effect, thus improving the catalytic performances of the samples [12,25]. As shown in Figure 3c,d, the shapes of the isotherms and pore distribution curves of the regenerated samples do not change much, and the S_BET_ and V_meso_ slightly decrease compared with those of the fresh TS-RH-2.5 sample (Table 1). These results indicate that the pore structures of the regenerated samples remain well after the recycle tests.

**Figure 4:**
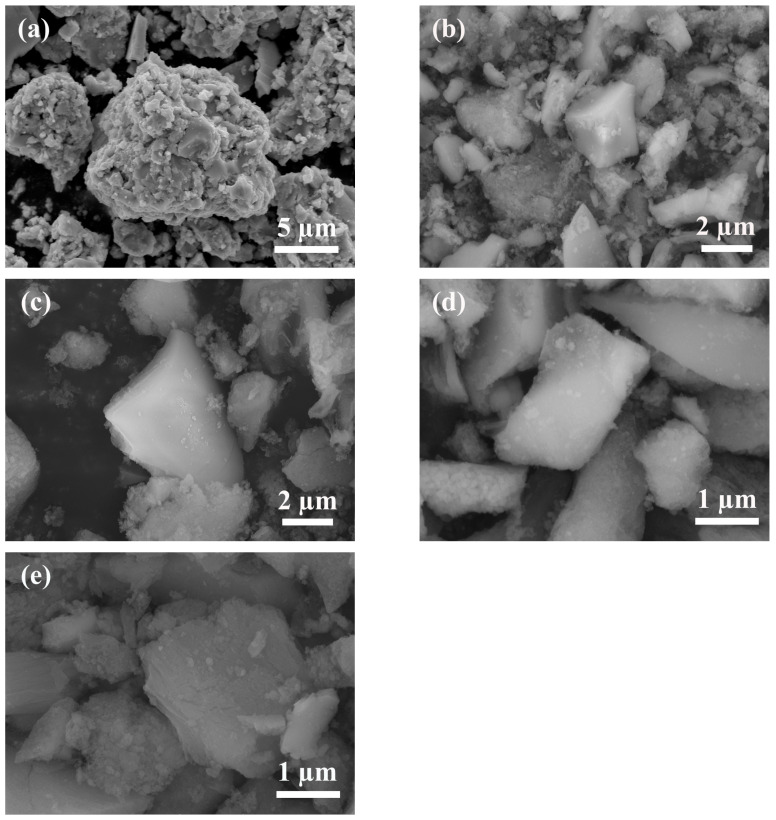
SEM images of the samples: (**a**) TiO_2_, (**b**) SiO_2_-RH, (**c**) TS-RH-2.5, (**d**) TS-RH-2.5c regenerated from the 15th ODS reaction of DBT, and (**e**) TS-RH-2.5c regenerated from the 15th ODS reaction of 4,6-DMDBT.

**Figure 5:**
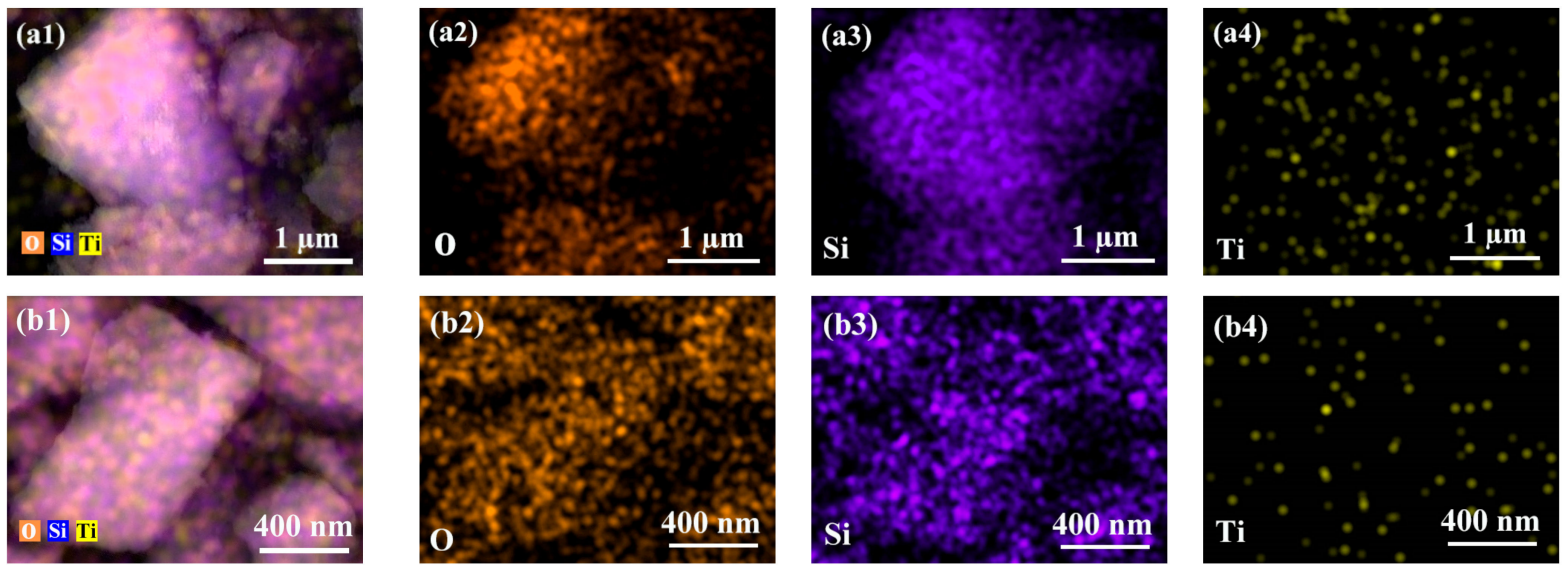

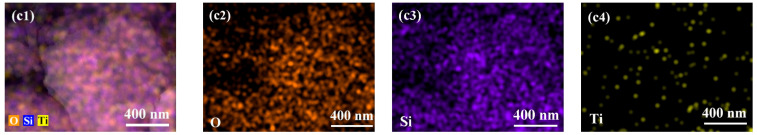
EDS elemental mappings of the samples: (**a**) TS-RH-2.5, (**b**) TS-RH-2.5c regenerated from the 15th ODS reaction of DBT, and (**c**) TS-RH-2.5c regenerated from the 15th ODS reaction of 4,6-DMDBT.

Figure 4 shows the SEM images of the SiO_2_-RH, pure TiO_2_, TS-RH-2.5, and TS-RH-2.5c samples. The pure TiO_2_ sample exhibits an uneven morphology, which is an aggregated structure composed of micrometer-sized grains (Figure 4a). The SiO_2_-RH carrier consists of particles of different sizes and no specific shape, with a maximum particle size of about 7 µm (Figure 4b). After TiO_2_ loading, there is little change in the morphology of the TS-RH-2.5 sample (Figure 4c). Compared with TS-RH-2.5, the morphology of the regenerated samples also does not change much after the recycle tests (Figure 4d,e). As can be seen from Figure 5, the Ti, Si, and O elements of all the samples are uniformly distributed over the entire surface, indicating that the TiO_2_ grains are relatively uniformly dispersed on the surface of the SiO_2_-RH carrier. This result is consistent with the results of XRD. Compared with the fresh TS-RH-2.5, there is no significant decrease in the number of Ti spots in the TS-RH-2.5c sample regenerated from 15th ODS reaction of 4,6-DMDBT, while the number of Ti spots in the TS-RH-2.5c sample regenerated from the 15th ODS reaction of DBT is slightly reduced. These results indicate that the TiO_2_ loadings of the regenerated samples decrease insignificantly after the recycle tests.

**Figure 6:**
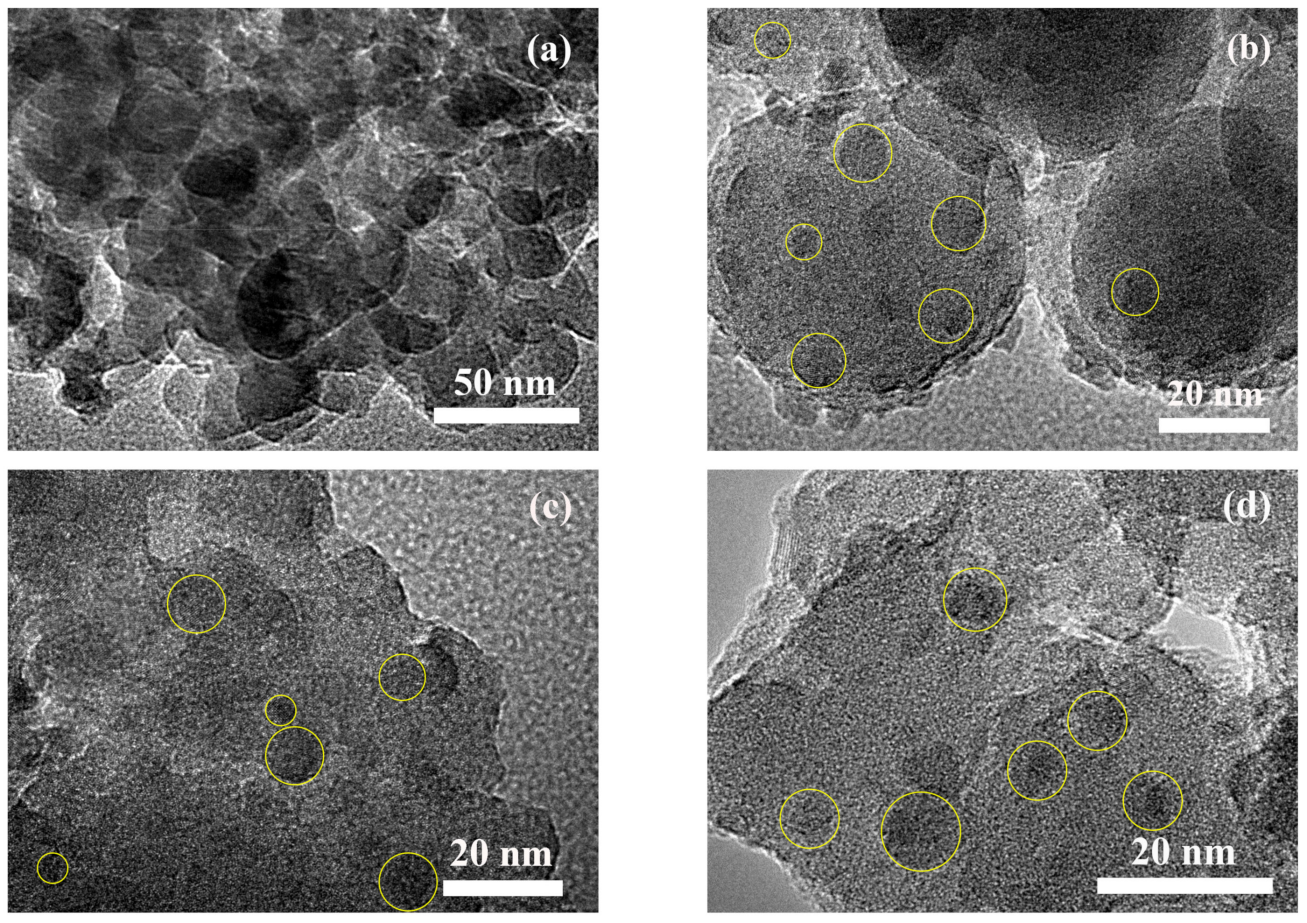
TEM images of the samples: (**a**) SiO_2_-RH, (**b**) TS-RH-2.5, (**c**) TS-RH-2.5c regenerated from the 15th ODS reaction of DBT, and (**d**) TS-RH-2.5c regenerated from the 15th ODS reaction of 4,6-DMDBT.

**Figure 7:**
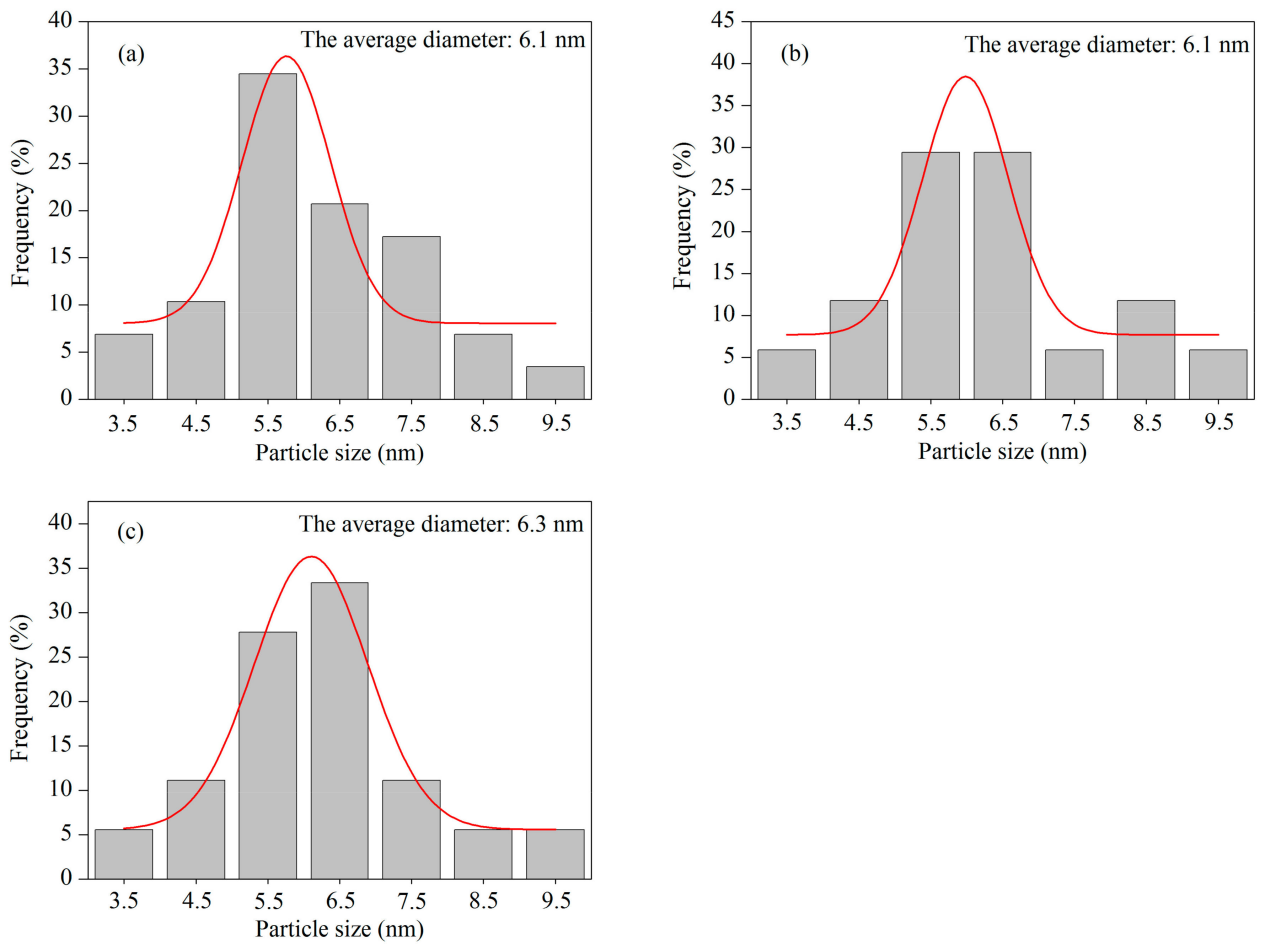
Particle size distribution histograms of the samples: (**a**) TS-RH-2.5, (**b**) TS-RH-2.5c regenerated from the 15th ODS reaction of DBT, and (**c**) TS-RH-2.5c regenerated from the 15th ODS reaction of 4,6-DMDBT.

Figure 6 and Figure 7 show TEM pictures and histograms of the particle size distributions of the samples, respectively. As shown in Figure 6a, the SiO_2_-RH sample has a large number of mesopores, which is consistent with the results of the N_2_ adsorption–desorption isotherms. After TiO_2_ loading, some TiO_2_ grains with an average diameter of 6.1 nm appear in the TS-RH-2.5 sample, which are dispersed relatively uniformly on the surface of the SiO_2_-RH carrier (Figure 6b, yellow circles, and Figure 7a). After the 15th run, nanoscale TiO_2_ grains are still observed on the SiO_2_-RH carrier and remain uniformly distributed (Figure 6c,d, yellow circles). In addition, the average diameters of the TiO_2_ grains in the TS-RH-2.5c samples regenerated from the 15th ODS reactions of DBT and 4,6-DMDBT are 6.1 and 6.3 nm, respectively (Figure 7b,c). These results indicate that the TiO_2_ grains do not agglomerate during multiple calcination and regeneration, resulting in a good catalytic stability of the TS-RH-2.5 sample. 

### 2.2. Catalytic Performance on Oxidative Desulfurization

**Figure 8:**
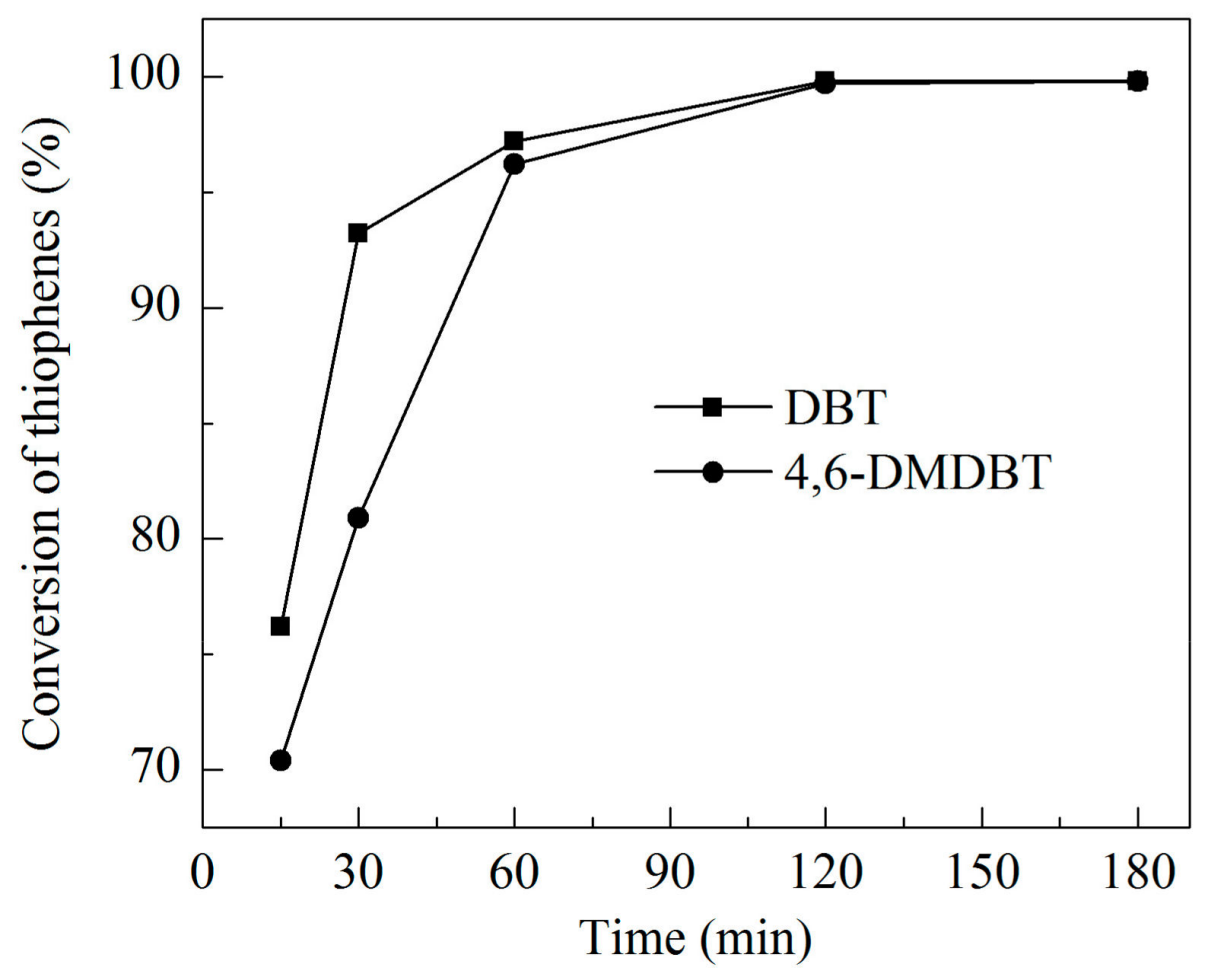
Effect of reaction time on the ODS of DBT and 4,6-DMDBT model oils catalyzed by the TS-RH-2.5 sample. Reaction conditions: model oils, 10 mL; catalyst to oil ratio, 10.0 g/L; TBHP/S molar ratio, 2.1; and temperature, 333 K.

The catalytic performances of the TS-RH-x samples are investigated using the ODS reactions of DBT and 4,6-DMDBT model oils as the probe reactions, and the results are shown in Table 1. When the TiO_2_ loading is 0.5%, the conversions of DBT and 4,6-DMDBT are 97.7% and 96.6%, respectively. As the TiO_2_ loading increases from 0.5 to 2.5%, the conversions of DBT and 4,6-DMDBT increase. Continuing to increase the TiO_2_ loading, the conversions of DBT and 4,6-DMDBT do not change much. Therefore, the more suitable TiO_2_ loading is 2.5%. Figure 8 shows the effect of reaction time on the ODS performance of the TS-RH-2.5 sample. It can be seen that, as the reaction time is prolonged from 15 min to 2 h, the conversions of DBT and 4,6-DMDBT increase linearly. Continuing to prolong the reaction time, the conversions of DBT and 4,6-DMDBT remain basically unchanged. Therefore, the more suitable reaction time for both the DBT and 4,6-DMDBT model oils is 2 h. 

In order to investigate the reason for the good catalytic performance of the TS-RH-2.5 sample in the ODS of DBT and 4,6-DMDBT model oils, several blank reactions were designed and carried out under the reaction conditions in Table 1. When the catalyst was not involved in the reaction, the conversions of DBT and 4,6-DMDBT were 21.8 and 15.1%, respectively, indicating that the oxidizing effects of TBHP on DBT and 4,6-DMDBT were poor. When TS-RH-2.5 was used as the catalyst and no oxidant was added, the conversions of DBT and 4,6-DMDBT were 20.0% and 14.0%, respectively, indicating that the TS-RH-2.5 sample had a certain adsorption desulfurization effect, but this effect was poor [15]. Compared to the results of the catalyst blank experiment, the conversions of DBT and 4,6-DMDBT increased slightly when the SiO_2_-RH and pure TiO_2_ were used as the catalysts (see Table 1), indicating that the SiO_2_-RH and pure TiO_2_ were poorly active. Based on the above characterization results, as well as the results of the blank experiments, the good catalytic oxidative desulfurization performance of TS-RH-2.5 can be attributed to (1) its high specific surface area and mesopore volume and (2) the uniform dispersion of TiO_2_ grains with an average diameter of 6.1 nm on the surface of the mesoporous SiO_2_-RH carrier, which greatly increases the active sites of the sample, consequently improving its catalytic activity. 

**Figure 9:**
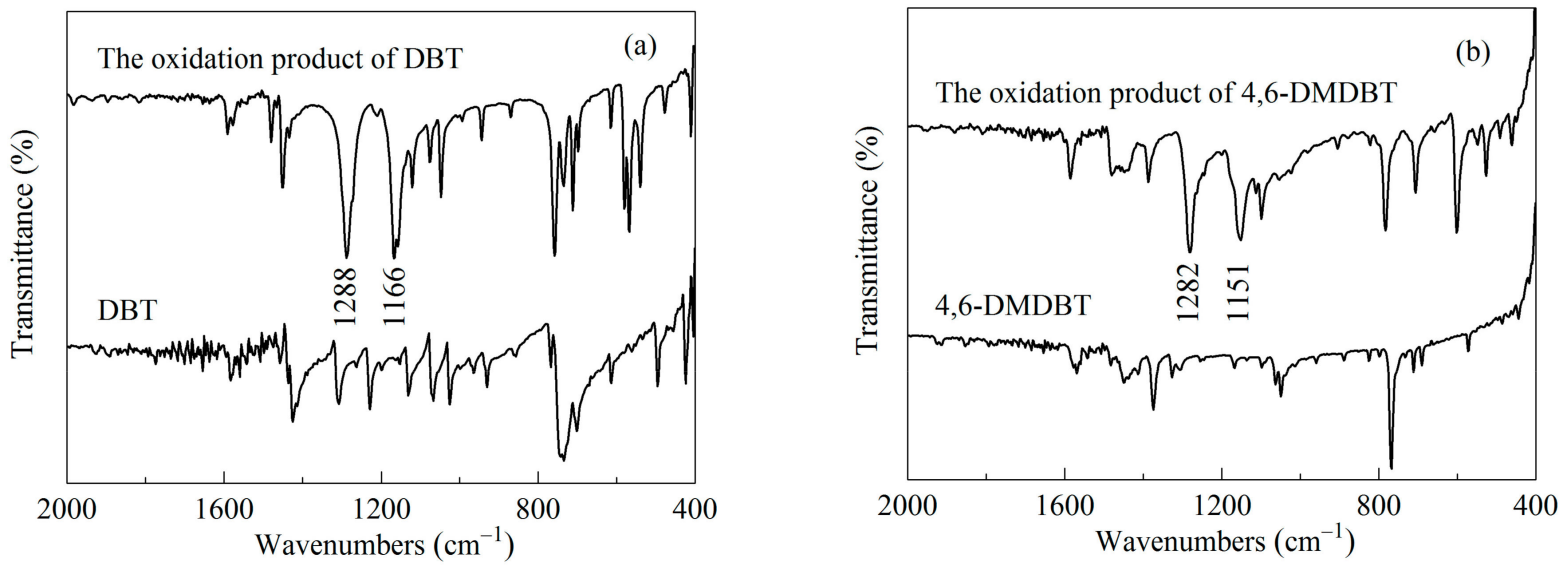
FT-IR spectra of the reactants and their corresponding products: (**a**) DBT and its oxidation product and (**b**) 4,6-DMDBT and its oxidation product.

**Figure 10:**
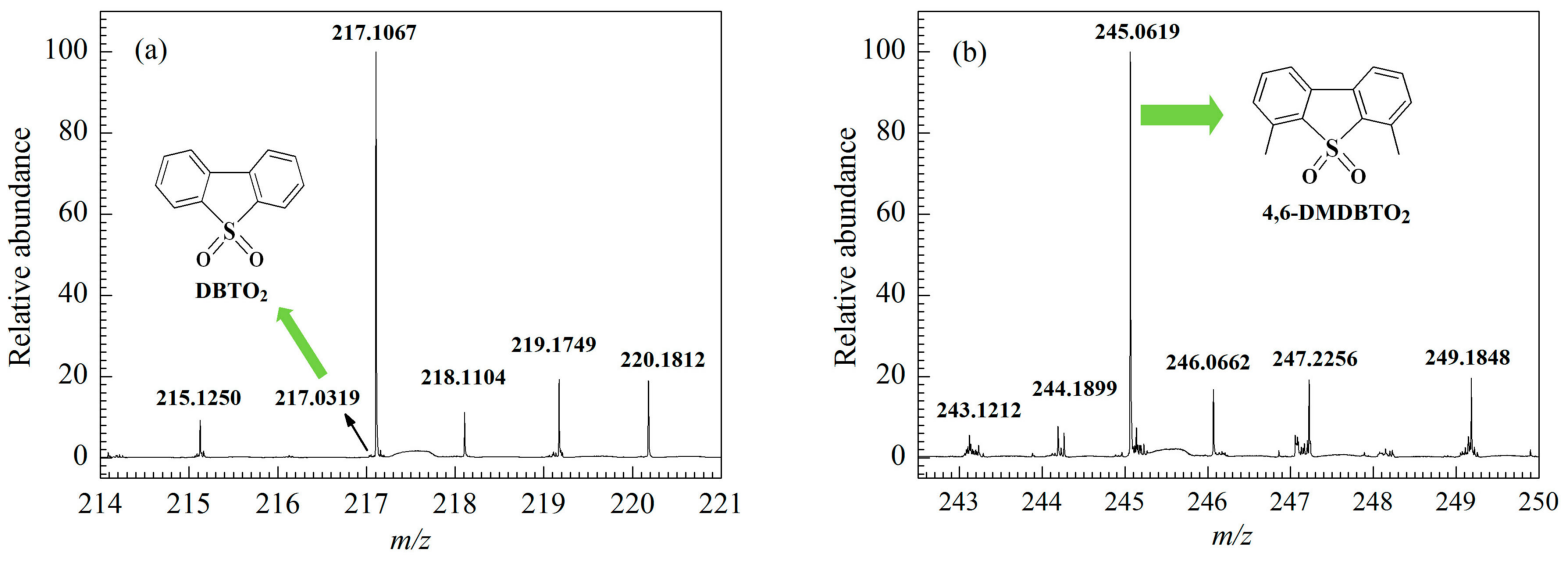
LC-MS spectra of the oxidation products of DBT (**a**) and 4,6-DMDBT (**b**).

The oxidation products of DBT and 4,6-DMDBT were analyzed by the means of the FT-IR and LC-MS techniques. As shown in Figure 9a, compared with DBT, two new absorption peaks at 1166 and 1288 cm^−1^ appear in the oxidation product of DBT, which can be attributed to the symmetric and asymmetric stretching vibrations of the sulfone (O=S=O), respectively [26,27]. Similarly, the 4,6-DMDBT oxidation product presents new absorption peaks at 1151 and 1282 cm^−1^ (Figure 9b), which can be attributed to the symmetric and asymmetric stretching vibrations of the sulfone (O=S=O), respectively [26,27]. As shown in Figure 10, the ion peak at the mass to charge ratio (*m*/*z*) of 217.0319 matches well with the *m*/*z* of the dibenzothiophene sulfone (DBTO_2_) quasi-molecular ion ([M+H]^+^, 217.0318). Moreover, the ion peak at the *m*/*z* of 245.0619 has a good match with the *m/z* of the 4,6-dimethyldibenzothiophene sulfone (4,6-DMDBTO_2_) quasi-molecular ion ([M+H]^+^, 245.0631). The above results demonstrate that the oxidation products of DBT and 4,6-DMDBT are DBTO_2_ and 4,6-DMDBTO_2_, respectively. It is worth noting that the presence of sulfoxide is not detected in the FT-IR and LC-MS characterizations of the oxidation products of DBT and 4,6-DMDBT. This result indicates that sulfoxide would be very unstable in this reaction system and is further oxidized to the corresponding sulfone, which is consistent with the results reported in the literature [8,28]. 

**Figure 11:**
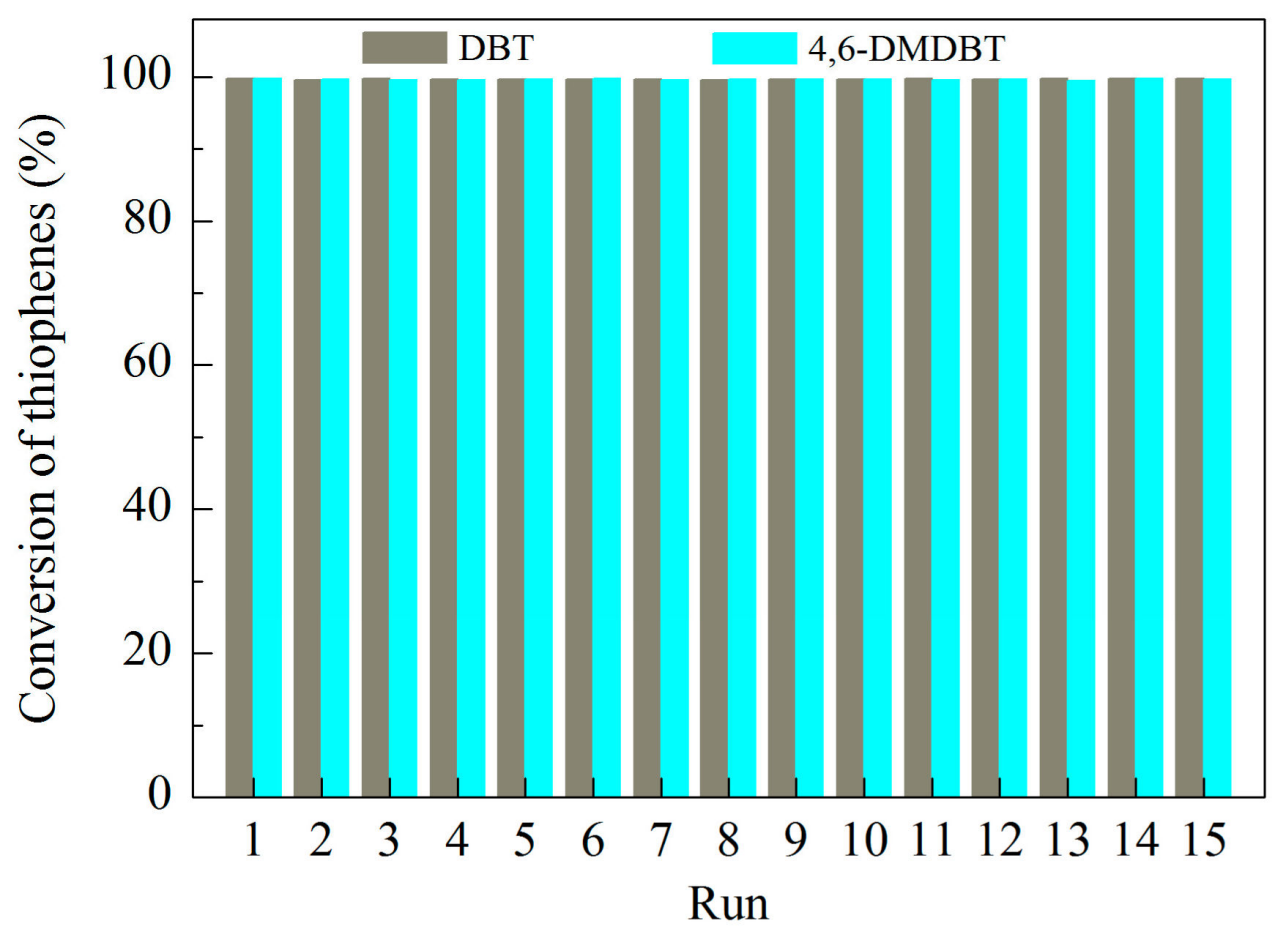
Recycle tests for the ODS of DBT and 4,6-DMDBT model oils over the TS-RH-2.5c samples.

Figure 11 shows the results of the cyclic experiments for the TS-RH-2.5 sample. As one can see from Figure 11, after the 15th run, the conversion of DBT remains as high as 99.8%, and the conversion of 4,6-DMDBT remains as high as 99.7%. The samples before and after the recycle tests were characterized by the means of the XRD, UV-Vis, N_2_ adsorption–desorption isotherms, SEM, TEM, and ICP-OES techniques. The results of XRD (Figure 1b) indicate that the regenerated samples still have an amorphous structure compared to the fresh sample. The results of UV-Vis (Figure 2b) indicate that the regeneration of the spent catalysts using the calcinating method is effective in removing the adsorbed oxidized products from the catalysts, and the reduction in the TiO_2_ loading of the regenerated samples is not significant compared to the fresh sample. The results of the N_2_ adsorption–desorption isotherms (Figure 3c,d, Table 1) indicate that the pore structures of the regenerated samples are well-maintained after the recycle tests. The results of SEM and EDS (Figure 4 and Figure 5) demonstrate that the morphology of the regenerated samples remains unchanged, the distribution of the TiO_2_ grains on the surface of the SiO_2_-RH carrier remains relatively uniform, and the loading of TiO_2_ decreases insignificantly. The results of TEM (Figure 6 and Figure 7) suggest that the nano-sized TiO_2_ grains still remain uniformly distributed on the SiO_2_-RH carrier without agglomeration after the recycle tests. The results of ICP-OES show that the TiO_2_ loadings of TS-RH-2.5, TS-RH-2.5c regenerated from the 15th ODS reaction of DBT, and TS-RH-2.5c regenerated from the 15th ODS reaction of 4,6-DMDBT are 2.55, 2.21, and 2.28%, respectively. These results indicate that the loss of TiO_2_ during the recycle tests is negligible. The above results indicate that the TS-RH-2.5 sample has a good catalytic activity and recycling performance. In addition, as shown in Table 2, compared with the TiO_2_-based catalysts reported in the literature [14,15,16,29,30,31,32], the recycling performance of the TS-RH-2.5 sample prepared in this work is comparable or better than those of the reported samples. 

### 2.3. Reaction Mechanism of the ODS Process

**Figure 12:**
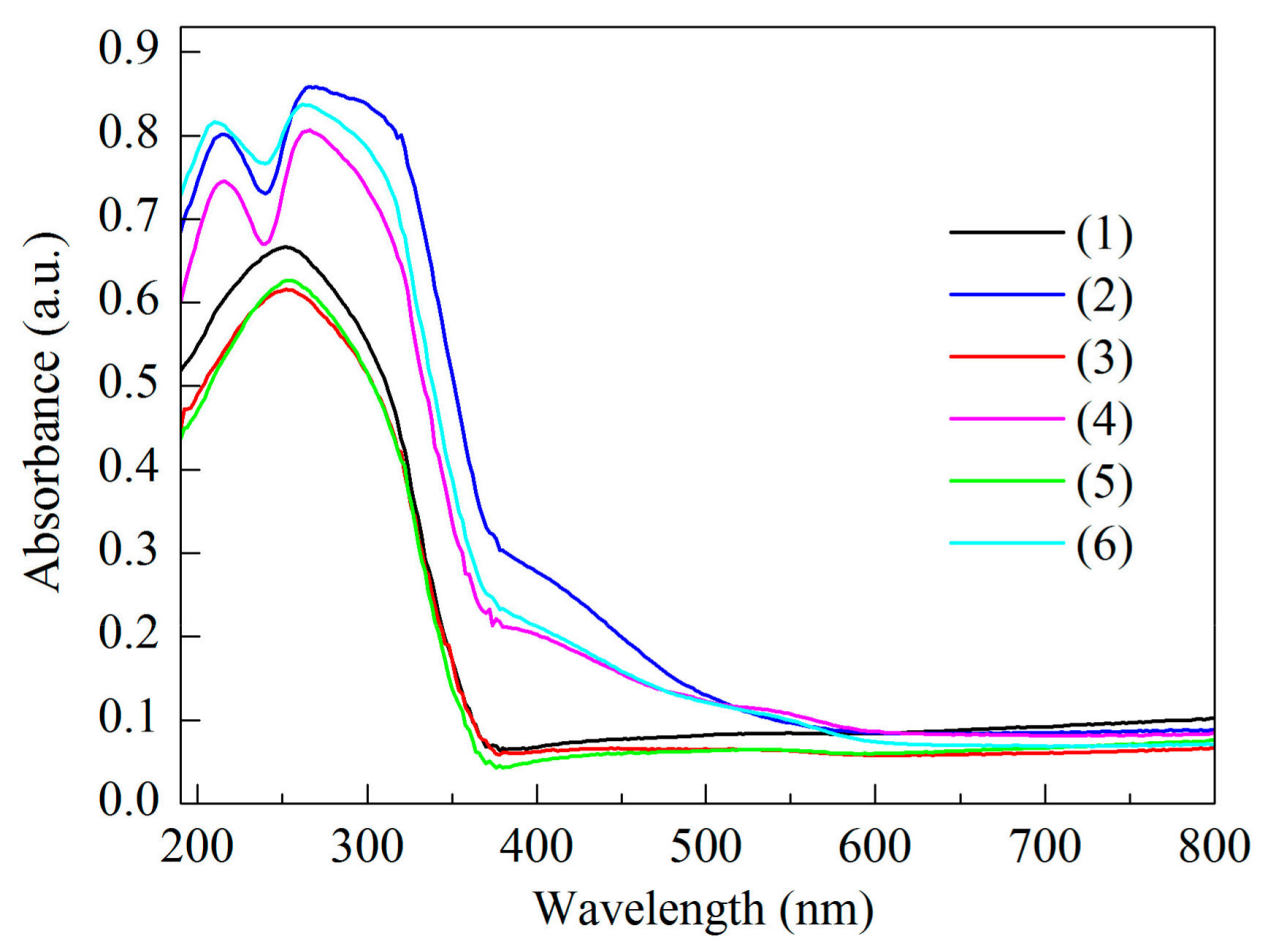
UV-Vis spectra of fresh TS-RH-2.5 and TS-RH-2.5c samples: (1) fresh TS-RH-2.5, (2) fresh TS-RH-2.5 treated with TBHP, (3) TS-RH-2.5c regenerated from the 15th ODS reaction of DBT, (4) TS-RH-2.5c regenerated from the 15th ODS reaction of DBT and treated with TBHP, (5) TS-RH-2.5c regenerated from the 15th ODS reaction of 4,6-DMDBT, and (6) TS-RH-2.5c regenerated from the 15th ODS reaction of 4,6-DMDBT and treated with TBHP.

Similar to our previously reported method [7], the samples were treated with an aqueous solution of TBHP and detected by the means of the UV-Vis technique, allowing for the capture of Ti-peroxo species. The color of the TS-RH-2.5 sample changed from white to light yellow after the treatment with the TBHP aqueous solution, and a clear absorption peak appeared at 370–600 nm (see Figure 12), which can be attributed to the charge transfer transition of the peroxo ligand to the titanium center in the Ti-peroxo species, indicating that the Ti-peroxo species (Ti-OO^t^Bu) was produced [7,31,32,33]. Generally, the Ti-peroxo species is considered to be the active intermediate in the catalytic oxidation of various organic substrates in catalytic systems containing TiO_2_-based catalysts and hydroperoxide [7,31,32,33]. In addition, the TS-RH-2.5c samples regenerated after the 15th recycle test were subjected to the same treatment, and both of them showed significant absorption peaks at 370–600 nm, indicating that the regenerated samples still had a strong ability to bind TBHP to form the Ti-OO^t^Bu species. This result is in agreement with the results of the cyclic experiments. 

**Scheme 1:**
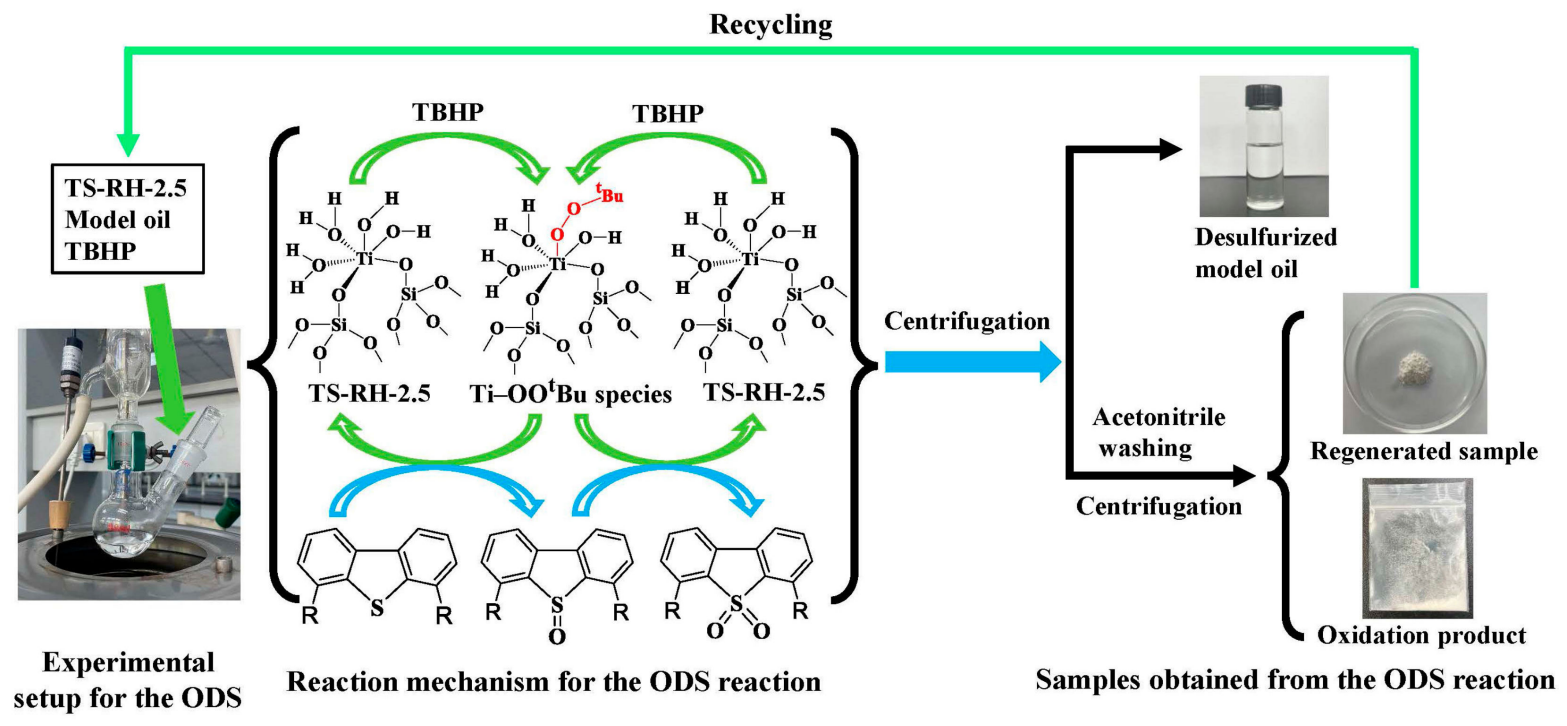
Schematic representation of the reaction mechanism and process flow for the oxidative removal of DBT and 4,6-DMDBT in the TS-RH-2.5 and TBHP catalytic system (R represents H or CH_3_).

The UV-Vis results indicate that the TS-RH-2.5 sample contains tetra-coordinated titanium species, hexa-coordinated titanium species, and anatase-phase TiO_2_. Among them, the hexa-coordinated titanium species is the main component. Generally, the silicon in amorphous SiO_2_ is interconnected in a silico-oxygen tetrahedron manner. Based on the results of XRD and UV-Vis, as well as the properties of amorphous TiO_2_/SiO_2_ [23,24], the structural schematic of the TS-RH-2.5 sample represented by hexa-coordinated titanium species can be illustrated as shown in Scheme 1. From the Scheme 1, it can be seen that the titanium atoms not only bind to two silico-oxygen tetrahedrons through oxo-bridges, but also bind to two water molecules and two hydroxyl groups. 

Based on the results of Figure 9, Figure 10 and Figure 12, the reaction mechanism and process flow for the oxidative removal of DBT and 4,6-DMDBT in the TS-RH-2.5 and TBHP catalytic system are proposed, as shown in Scheme 1. First, the catalyst, model oil, and TBHP are fed in a set ratio. The reaction starts with the nucleophilic attack of TBHP on the titanium active sites to form the active intermediate Ti-OO^t^Bu species. Then, the sulfur atoms in DBT (or 4,6-DMDBT) nucleophilically attack the Ti-OO^t^Bu species to generate the sulfoxides, while releasing the TS-RH-2.5 catalyst. The sulfoxides are very unstable in this reaction system and are quickly oxidized by the Ti-OO^t^Bu species to form the corresponding sulfones [8,28]. In this reaction system, the by-product is tert-butanol [28]. Since the sulfones are insoluble in n-octane, the products and catalysts precipitate out of the reaction medium immediately after the reaction. Therefore, at the end of the reaction, a simple filtration or centrifugation is all that is required to obtain the desulfurized model oil and the solid. The solid is centrifuged and washed by hot acetonitrile to obtain the supernatant and the remaining solid. Then, the supernatant is concentrated by rotary evaporation and vacuum-dried to obtain the oxidized product. The remaining solid is then dried and calcined to obtain the regenerated sample. Finally, the regenerated sample can be recycled for cycling experiments of the ODS. The whole ODS process is simple, mild, and easy to control, which is a beneficial supplement to the current industrial hydrodesulfurization process. 

## 3. Materials and Methods

### 3.1. Materials

RH originated from the College of Agriculture of Yangtze University (Jingzhou, China). Tetrabutyl titanate (TBOT, 97%) and Tert-butyl hydroperoxide (TBHP, ~5.5 mol/L in decane) were purchased from Sigma Aldrich (Shanghai, China) Trading Co., Ltd. Dibenzothiophene (DBT, 99%), 4,6-dimethyldibenzothiophene (4,6-DMDBT, 97%), and TBHP solution (70% in H_2_O) were purchased from Shanghai Aladdin Biochemical Technology Co., Ltd. (Shanghai, China). Isopropanol (IPA), hydrochloric acid, n-octane, acetonitrile, ethanol, and other reagents (A.R.) were purchased from Sinopharm Chemical Reagent Co., Ltd. (Shanghai, China).

### 3.2. Synthesis of TiO_2_/SiO_2_ Samples

The RH was pretreated with hydrochloric acid according to the method reported by our team [12] to obtain pretreated RH powder. About 20.0 g of the pretreated RH powder was dried at 383 K for 4 h to obtain the absolutely dried RH powder. About 5.0 g of the absolutely dried RH powder was calcined at 873 K for 5 h to obtain the SiO_2_ powder in RH, which was denoted as SiO_2_-RH. Accordingly, the mass fraction of SiO_2_-RH in the absolutely dried RH powder can be calculated as 19.6 wt.%. TiO_2_/SiO_2_ catalysts were prepared by the incipient wetness impregnation method using the absolutely dried RH powder as both the mesoporous template and silicon source [12]. A typical preparation operation was as follows: first, 10.0 g of RH powder was placed in a 100 mL beaker. Then, TBOT was dissolved in 10.3 mL of IPA according to the mass ratio of TiO_2_:SiO_2_ = x:100 to obtain a light yellow clear solution. The clear solution was added drop by drop to the RH powder with constant stirring until a saturated impregnation state was achieved. Then, the mixture was aged at room temperature for about 24 h and dried at 383 K for 8 h. Finally, white powder was obtained by grinding and calcining at 873 K for 5 h (1 K/min). It was labeled as TS-RH-x, where ‘x’ represents the loading of TiO_2_. Using a similar method, TS-RH-0.5, TS-RH-1.0, TS-RH-5.0, and TS-RH-10 samples were prepared. 

As a comparison, TiO_2_ was prepared by direct calcination. Typically, about 4.0 g of TBOT was placed in an evaporating dish and hydrolyzed naturally in the air at room temperature for 24 h to obtain a white solid. Then, the white solid was dried at 383 K for 6 h. Finally, the white solid was ground and calcined at 873 K for 4 h to obtain TiO_2_ powder.

### 3.3. Treatment of Samples with TBHP Aqueous Solution

The samples were treated with a 70% aqueous solution of TBHP according to the method reported by our team [7]. Typically, 50 mg of the TS-RH-2.5 sample was placed in a 25 mL beaker and 0.5 mL of TBHP aqueous solution was added dropwise. The mixture was stirred thoroughly with a glass rod to achieve a saturated impregnation state. Finally, the mixture was dried in a fume hood at room temperature for about 3 h, and a light yellow powder was obtained.

### 3.4. Characterization

The XRD patterns were recorded with Cu-Kα radiation on a Panalytical Empyrean X-ray diffractometer (Malvern Panalytical Ltd., Malvern, UK) with a scanning angle (2θ) of 10–80°. The UV-Vis spectra were measured by a Lambda 650S spectrometer (PerkinElmer Inc., Shelton, CT, USA) with a spectral range of 190–800 nm. The FT-IR spectra were measured by a Nicolet 6700 infrared spectrometer (Thermo Fisher Scientific Inc., Waltham, MA, USA) with a pure KBr background. Measurements were made over a wavelength range of 400–4000 cm^−1^. The SEM images and elemental maps were taken by a TESCAN MIRA3 field emission scanning electron microscope (TESCAN ORSAY Holding, a.s., Brno, Kohoutovice, Czech Republic) equipped with an XFlash Detector 630M energy dispersive spectrometer (EDS, Bruker Nano GmbH, Berlin, Germany). The TEM images were collected by an FEI TECNAI F20 transmission electron microscope (FEI Inc., Hillsboro, OR, USA). The elemental contents of the samples were determined by a PerkinElmer Optima 8000 inductively coupled plasma emission spectrometer (PerkinElmer Inc., Shelton, CT, USA). About 100 mg of the sample was placed in a high-pressure digestion jar, 2.0 mL of aqueous hydrofluoric acid (40%) was added, and low-temperature digestion (2 h at 323 K) was carried out in microwave digestion apparatus (MARS6, CEM Corporation, Matthews, NC, USA). After digestion, the solution was diluted and fixed in a plastic volumetric flask. Blanks were subjected to the same procedure. In addition, a mixed standard solution of silicon and titanium was prepared using standard stock solutions, the absorbances were measured at the set wavelengths, and the standard curves were drawn. The sample solutions were measured and their elemental contents were calculated from the standard curve. Two parallel samples were made for each sample and the final result was taken as the average of the two measurements. The N_2_ adsorption–desorption isotherms were carried out on a Micromeritics ASAP 2020HD88 physical adsorption instrument (Micromeritics Instrument Corporation, Norcross, GA, USA) at 77 K. Prior to analysis, all of the samples were degassed at 573 K for 4 h. The specific surface area (S_BET_) was determined by the BET equation. The cumulative pore volume (V_meso_) and pore size distribution were calculated by the Barrett–Joyner–Halenda (BJH) model from the desorption branch of the N_2_ physisorption isotherms. The absorption spectra and standard curves of the DBT and 4,6-DMDBT model oils were determined by a TU–1900 UV-Vis spectrophotometer (Beijing Puxi general instrument Co., Ltd., Beijing, China). The oxidized products of DBT and 4,6-DMDBT were analyzed by high-resolution liquid chromatography–mass spectrometry (LC-MS, Triple TOF 5600+, AB SCIEX Ltd., Framingham, MA, USA), a hybrid triple quadrupole time-of-flight mass spectrometer equipped with an ESI source operating in positive ion mode. 

### 3.5. Catalytic Activity and Recycle Tests

#### 3.5.1. Catalytic Activity

The selective oxidation reactions of DBT and 4,6-DMDBT were carried out in a 50 mL two-necked flask immersed in an oil bath and equipped with a condenser. The model oils of DBT and 4,6-DMDBT were prepared using n-octane as a solvent, and they all had a sulfur content of 1000 ppmw. Typically, 0.100 g of the TS-RH-2.5 catalyst and 10 mL of the DBT (or 4,6-DMDBT) model oil were added to the flask, and the mixture was stirred and heated to 333 K. The reaction was then initiated by adding 83 μL of TBHP (TBHP/S molar ratio of 2.1). The reaction was stopped after 2 h. The reaction mixture was cooled and centrifuged to obtain the oil phase and the solid. 

**Figure 13:**
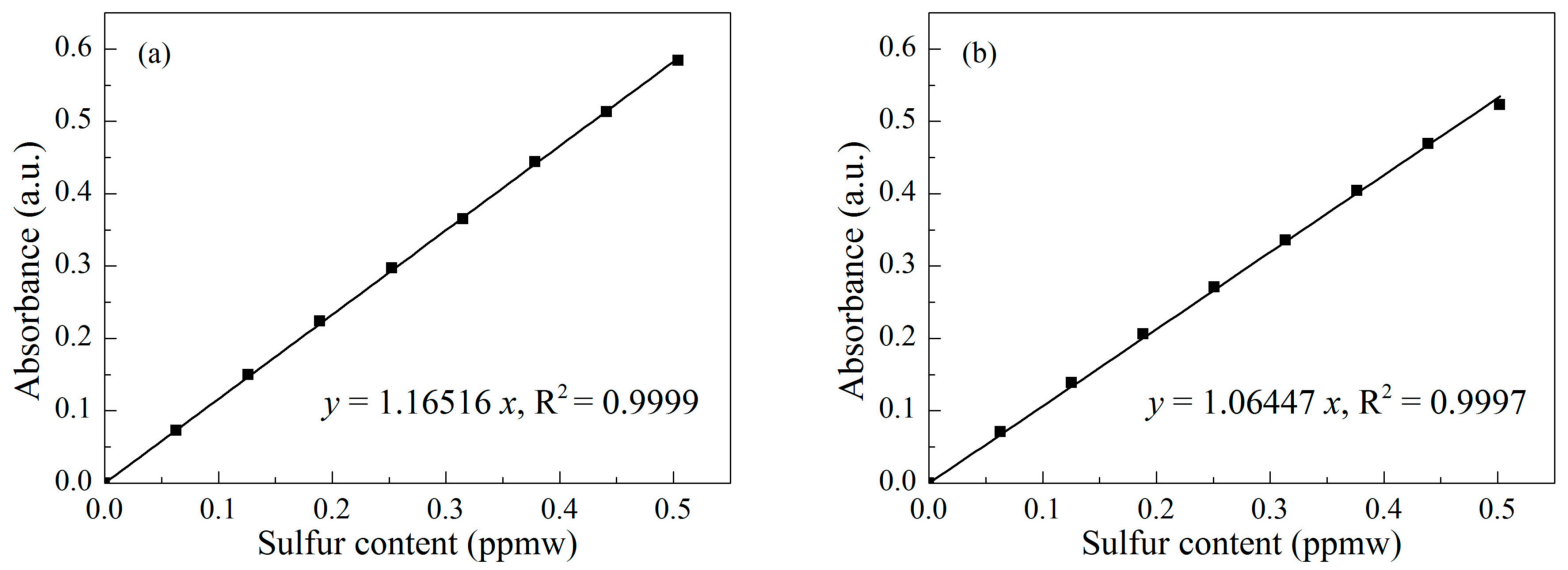
The standard curves of DBT (**a**) and 4,6-DMDBT (**b**) model oils.

The absorbance of the oil phase at 236 nm (241 nm for 4,6-DMDBT model oil) was determined on a TU-1900 UV-Vis spectrophotometer using ethanol as the diluent solvent and reference solution. The standard curve method was used to calculate the amount of DBT remaining in the oil phase, and the standard curves of the DBT and 4,6-DMDBT model oils are shown in Figure 13. As can be seen from Figure 13, the linear regression equation of the DBT model oil is *y* = 1.16516 *x*, with a correlation coefficient R^2^ of 0.9999 and a linear range of 0–0.504 ppmw. Similarly, the linear regression equation for the 4,6-DMDBT model oil is *y* = 1.06447 *x*, with a correlation coefficient R^2^ of 0.9997 and a linear range of 0–0.501 ppmw. The conversions of DBT and 4,6-DMDBT (*X*) were defined as the amount of the sulfide reacted divided by the initial amount of the sulfide added. 

**Figure 14:**
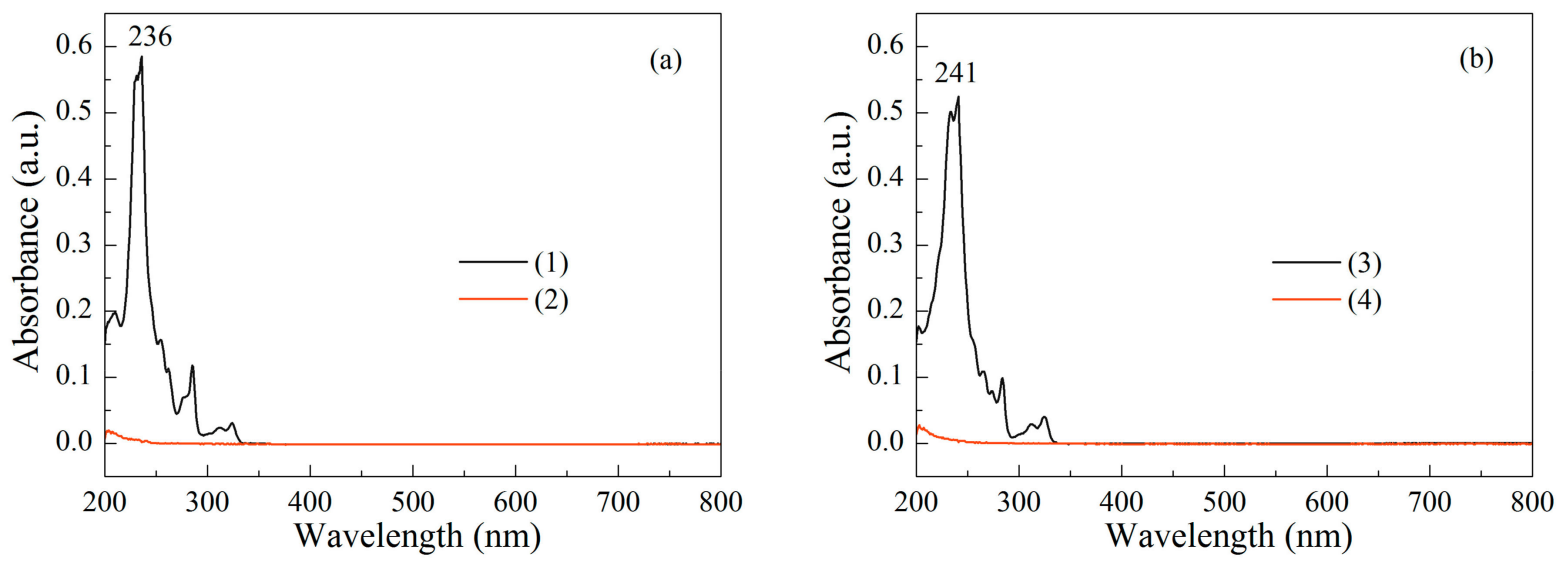
The absorption spectra of DBT (**a**) and 4,6-DMDBT (**b**) model oils before and after the ODS reaction catalyzed by the TS-RH-2.5 sample: (1) DBT model oil before the reaction diluted 2000 times, (2) DBT model oil after the reaction diluted 2000 times, (3), 4,6-DMDBT model oil before the reaction diluted 2000 times, and (4) 4,6-DMDBT model oil after the reaction diluted 2000 times.

Moreover, the DBT and 4,6-DMDBT model oils before and after the ODS reaction were diluted the same number of times (2000 times), and their absorption spectra were determined (see Figure 14). It is obvious from Figure 14 that the absorbances of the reacted DBT and 4,6-DMDBT model oils at the set wavelengths were greatly reduced to almost zero compared with before the reaction. These results indicate that the TS-RH-2.5 sample has high oxidative desulfurization activity.

In addition, the solid was centrifuged and washed several times with hot acetonitrile (323 K) to obtain the supernatant and the remaining solid. Then, the supernatant was concentrated on a rotary evaporator, and the residue was dried in a vacuum oven at room temperature for 24 h to obtain the oxidized product of DBT (or 4,6-DMDBT). Finally, the remaining solid was dried overnight at 383 K and calcined at 823 K for 2 h (1 K/min) to obtain the regenerated sample, which was labeled as TS-RH-xc. 

#### 3.5.2. Recycle Tests

Cycling experiments with the TS-RH-2.5 sample were performed in a 250 mL three-necked flask equipped with a condenser. The typical cyclic experimental procedure was similar to that in Section 3.5.1, with the following conditions for the first oxidation reaction: 150 mL of the DBT (or 4,6-DMDBT) model oil, 1.500 g of the catalyst, 1245 μL of TBHP, a temperature of 333 K, and a reaction time of 2 h. In each run, the dosages of TBHP and the model oil were calculated based on the ratio of catalyst to oil (10.0 g/L), the molar ratio of TBHP/S (2.1), and the mass of the catalyst obtained in the previous run. 

## 4. Conclusions

In summary, a series of mesoporous TS-RH-x samples were prepared using the incipient wetness impregnation method with RH as both the silicon source and mesoporous template. The samples were characterized more systematically using a series of characterization techniques. The results showed that, when the loading of TiO_2_ was lower than 2.5%, the samples had an amorphous structure. When the loading of TiO_2_ was higher than 2.5%, TiO_2_ in the anatase phase appeared in the samples. Compared with the pure TiO_2_, the TS-RH-x samples had a high S_BET_ and V_meso_. For the TS-RH-2.5 sample, TiO_2_ grains with an average diameter of 6.1 nm were uniformly dispersed on the surface of the mesoporous SiO_2_-RH carrier, which greatly increased the active sites of the TS-RH-2.5 sample, thus improving its catalytic activity. As a result, the TS-RH-2.5 sample exhibited high catalytic activity in the ODS of the DBT and 4,6-DMDBT model oils. The recycling performances of the TS-RH-2.5 sample were further investigated and the samples before and after the recycle tests were characterized. The results showed that the TS-RH-2.5 sample still maintained high DBT conversion (99.8%) and 4,6-DMDBT conversion (99.7%) rates without inactivation after the 15th run. The relevant characterization results showed that the crystal structure, pore structure, and morphology of the samples were well-maintained after the recycle tests. Using the UV-Vis technique, the Ti-OO^t^Bu species, the active intermediate of the ODS reaction, was successfully captured. In addition, a possible mechanism for the ODS of bulky organic sulfides over TS-RH-2.5 was proposed. The TS-RH-2.5 sample had a good structural stability and recycling performance, and it will be well applied in the deep oxidative desulfurization of fuel oils.

## Figures and Tables

**Table 1:** The pore structure parameters and catalytic performances of the samples.

Sample	S_BET_ (m^2^/g)	V_meso_ (cm^3^/g)	*X*_DBT_ (%)	*X*_4,6-DMDBT_ (%)
SiO_2_-RH	294.4	0.39	33.9	24.8
Pure TiO_2_	3.3	0.01	32.3	22.6
TS-RH-0.5	286.6	0.38	97.7	98.1
TS-RH-1.0	281.5	0.38	98.8	98.7
TS-RH-2.5	277.6	0.38	99.8	99.7
TS-RH-5.0	271.0	0.37	99.7	99.8
TS-RH-10	264.8	0.37	99.8	99.8
TS-RH-2.5c ^a^	246.9	0.34	-	-
TS-RH-2.5c ^b^	229.5	0.34	-	-

Reaction conditions: model oils, 10 mL; catalyst to oil ratio, 10.0 g/L; TBHP/S molar ratio, 2.1; temperature, 333 K; reaction time, 2 h. ^a^ Regenerated from the 15th ODS reaction of DBT; ^b^ Regenerated from the 15th ODS reaction of 4,6-DMDBT.

**Table 2:** Comparison of the TS-RH-2.5 sample in the ODS of DBT and 4,6-DMDBT model oils with the reported similar catalysts.

Sample	Temp. (K)	S Content (ppm)	Cyclic Number	*X*_DBT_ ^a^ (%)	*X*_4,6-DMDBT_ ^a^ (%)	Ref.
TS-0.2	333	1000, 500 ^b^	5	99.0	99.8	[14]
TS-50	353	500	2	100.0	–	[15]
TS-4/CM-β-CD	298	500	3	99.0	–	[16]
MT-1:4	373	500	8	94.6	–	[29]
TS-4	298	500	3	98.0	–	[30]
MIL-125-TRO	333	250	3	–	92.3	[31]
H-TiNTs	313	320	4	~98.0	–	[32]
TS-RH-2.5	333	1000	15	99.8	99.7	This work

^a^ The catalytic results of the last cyclic reaction of DBT and 4,6-DMDBT, respectively; ^b^ The sulfur contents of DBT and 4,6-DMDBT model oils are 1000 and 500 ppmw, respectively.

## Data Availability

The original contributions presented in the study are included in the article, further inquiries can be directed to the corresponding author.
